# Recent Advances in Cell Adhesive Force Microscopy

**DOI:** 10.3390/s20247128

**Published:** 2020-12-12

**Authors:** Ying Tu, Xuefeng Wang

**Affiliations:** 1 Department of Physics and Astronomy, Iowa State University, Ames, IA 50011, USA; yingtu@iastate.edu; 2 Molecular, Cellular, and Development Biology Interdepartmental Program, Iowa State University, Ames, IA 50011, USA

**Keywords:** integrin, cell adhesion, cell adhesive force, cell traction force, integrin tension, tension sensor, DNA, cellular force imaging, super-resolution, mechanotransduction, mechanobiology

## Abstract

Cell adhesive force, exerting on the local matrix or neighboring cells, plays a critical role in regulating many cell functions and physiological processes. In the past four decades, significant efforts have been dedicated to cell adhesive force detection, visualization and quantification. A recent important methodological advancement in cell adhesive force visualization is to adopt force-to-fluorescence conversion instead of force-to-substrate strain conversion, thus greatly improving the sensitivity and resolution of force imaging. This review summarizes the recent development of force imaging techniques (collectively termed as cell adhesive force microscopy or CAFM here), with a particular focus on the improvement of CAFM’s spatial resolution and the biomaterial choices for constructing the tension sensors used in force visualization. This review also highlights the importance of DNA-based tension sensors in cell adhesive force imaging and the recent breakthrough in the development of super-resolution CAFM.

## 1. Introduction

Cells are dynamic systems which constantly undergo biochemical and physical activities. Early cell biology studies have focused on understanding the biochemical aspects of cells, such as genetics, immunology, development, pathology, etc. The physical aspect received relatively less attention until recent decades. As cells live in a physical world, many biological motions in metazoan cells including cell adhesion, migration and division are driven by physical forces. These forces are critical for the normal functions and sustainability of cells. In particular, cell adhesive force, a result of cell–matrix and cell–cell biophysical interactions, has emerged as an important physical signal in regulating cell functions and maintaining tissue integrity. In the long term, cell adhesive force can also be converted to biochemical signals through mechanotransduction, and take effect on cell survival [1], proliferation [2], differentiation [3], cancer progression [4], biological development [5], immune response [6] and more. Hence, substantial research interests have been drawn to study cell adhesive force and its related biological functions in the recent four decades.

**Figure 1:**
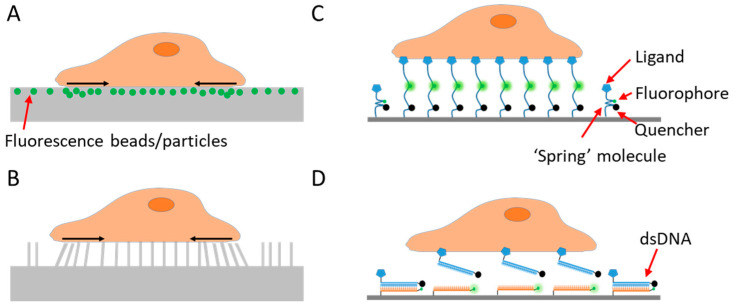
Schematics of techniques for cell adhesive force imaging. (**A**) Traction force microscopy (TFM) using an elastic polymer film doped with fluorescence beads or particles as the substrate. TFM converts cell adhesive force to substrate deformation. (**B**) TFM using micropillar arrays as the substrate. TFM records the bending of the micropillar arrays and computes the cell adhesive force. (**C**) Fluorescent tension sensor (FTS) converts cell adhesive force to fluorescence using an “entropic spring” molecule. (**D**) FTS based on binary material with distinct on/off states such as double-stranded DNA (dsDNA) or hairpin DNA.

To study a signal, one needs first to detect and quantify the signal. As cell adhesive force is spatially distributed across the cell–matrix or cell–cell interface, visualization of the force would provide critical information about the force distribution and dynamics. Cell adhesive force visualization had not been easily accessible due to the miniscule level of force (at the piconewton (pN)–nanonewton (nN) level) produced by cells and the invisible nature of force signals. To “see” the force, one needs to find an approach to convert the invisible force to a visible signal. Retrospectively speaking, cell adhesive force sensing methods can be categorized into two types: force-to-strain conversion adopted in traction force microscopy (TFM), and force-to-fluorescence conversion adopted by fluorescent tension sensors (FTSs). The prototypes of TFM were developed in the 1980s. Cell adhesive force was first visualized by plating cells on ultrathin elastic silicone films [7]. The cells contracted and produced wrinkles on the film which can be imaged by an optical microscope. In the following decades, a series of TFMs have been developed based on this strategy of force-to-strain conversion [8,9,10,11,12,13,14,15,16]. The force–reporting substrates are generally elastic materials embedded with fluorescent markers reporting the force-induced substrate strains (Figure 1A), or with micro-patterns whose deformation can be monitored (Figure 1B). With computational modeling, the substrate strain maps are converted to cell stress maps, hence reporting the lateral distribution of cell adhesive force. TFM pioneered cell adhesive force visualization and has the unique advantages in measuring whole cell force.

However, compared to the cell structural imaging that benefits from fluorescent staining or fluorescent proteins, strain-based TFM has inferior resolution, which is usually in the range of microns [17]. As many force transmission structures in cells, such as podosomes, invadopodia, focal complexes, filopodia, etc., are in the size of microns or submicrons, new techniques were in demand to improve the resolution of cell adhesive force imaging. A new strategy for force imaging was developed in the recent decade. A number of techniques adopted force-to-fluorescence conversion to visualize cell adhesive force. Such a strategy was first adopted by tension sensors that are genetically expressed inside cells [18,19]. The strategy was then adopted by extracellular tension sensors that are immobilized on surfaces and target specific force-transmitting receptors on the cell membrane [20,21,22,23,24] (Figure 1C,D). These tension sensors are force-activatable units that emit fluorescence or alter their fluorescent properties under tension. The onsite fluorescent signal responding to local molecular force events gives rise to unprecedented spatial resolution and sensitivity in cell adhesive force imaging. Here we collectively dub these sensors as fluorescent tension sensors (FTSs). There are already several excellent reviews covering TFM and FTSs, separately [17,25,26,27,28]. This review compares the performance of TFM and FTSs, and focuses on the recent advances in spatial resolutions of these methods. This review also discusses biomaterial choices for constructing FTSs and highlights the performance of DNA and DNA-like materials as the FTS constructs. 

Of note, there are another series of point-based single-molecule force techniques such as atomic force microscopy (AFM) [29], optical tweezers [30], magnetic tweezers [31] and biomembrane force probes [32]. These techniques proactively apply force to probe the mechanical properties of single biomolecules or molecular bonds, and are advantageous in obtaining the force spectroscopy of biomechanical events such as protein unfolding, bond dissociation, etc. Because these techniques do not image force (AFM generally does not perform imaging when used as a force probing tool) and the force is produced by the instruments as a probing signal, we do not categorize them into CAFM in this review as CAFM is developed to image cell-generated force. Readers interested in these single-molecule force techniques may refer to other review articles [33]. 

## 2. Advances in Cell Adhesive Force Microscopy in Term of Spatial Resolution

In this review, we focus on presenting and discussing the spatial resolutions offered by the two branches of CAFMs: TFM and FTS-enabled CAFM. Table 1 summarizes their spatial resolutions. 

### 2.1. Traction Force Microscopy Pioneered Cell Adhesive Force Imaging 

**Figure 2:**
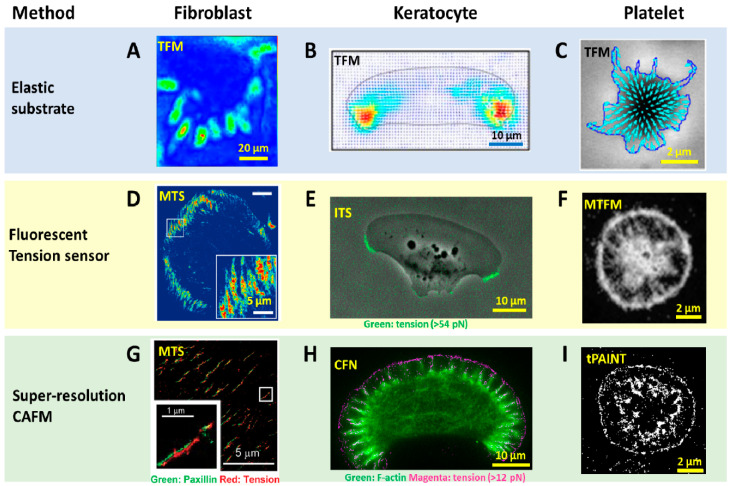
Advances in spatial resolution of cell adhesive force imaging techniques. (**A**–**C**) Force maps of a fibroblast [57], a migatory keratocyte [58] and a stationary platelet [59] obtained by TFM. The resolution is in the range of 1–5 µm. (**D**–**F**) Force maps of a fibroblast obtained by molecular tension sensor (MTS) [43], a keratocyte obtained by integrative tension sensor (ITS) [50] and a platelet obtained by molecular tension fluorescence microscopy (MTFM) [60], respectively. The resolutions are at the diffraction limit: 0.3–0.4 µm. (**G**–**I**) super-resolution force maps of a fibroblast obtained by MTS assisted with Bayesian computation [61], a keratocyte obtained by cellular force nanoscopy (CFN) [54] and a platelet obtained by tPAINT [55], respectively. Resolutions were reported as 50–100 nm for the live cell samples.

To detect and evaluate cell adhesive force, the prototype of traction force microscopy (TFM) was developed in the 1980s [7]. Subsequently, TFM was continuously improved and the algorithm was also refined for cell adhesive force computation. In TFM, cells are cultured on deformable polymer films [34] (Figure 1A) or micropillar arrays [10,35,36] (Figure 1B). The deformation of the substrates caused by cells is monitored by optical microscopy. To aid the deformation monitoring, the substrates are often embedded with fluorescent beads and the micropillar tips are fluorescently stained. By converting the displacements of the elastic substrates or micropillar arrays to traction stresses, TFM quantitatively maps force distribution at the interface between a cell and the extra-cellular matrix (ECM). TFM also has wide dynamic range and is adaptive to force measurement at different force levels. This can be achieved simply by altering substrate elasticity. However, because TFM computes cellular force from a strain map in an indirect manner, it had limited spatial resolution and sensitivity as an imaging technique. It is challenging for TFM to resolve forces in micron-sized cells (e.g., the small blood cell platelets) or subcellular structures (focal complexes, podosomes, filopodia, etc.). Figure 2A–C shows the representative TFM images of fibroblasts, keratocytes and platelets. 

Continuous efforts have been made to improve the resolution of TFM. A high-resolution TFM microscope compatible with spinning-disk confocal setting was developed in Waterman’s lab with a 10–20 µm thick fibronectin-coated polyacrylamide substrate [37]. The polymer substrates were embedded with a high density of fluorescent beads. By reconstructing the cell-induced bead displacements to cellular traction stress, a spatial resolution of ~1 μm was achieved using the Fourier-transform traction cytometry method. The improved spatial resolution was ascribed to high areal density (10 beads/μm^2^) of small-sized (40 nm) fluorescent beads in the polymer substrates. This high-resolution traction force microscopy revealed a robust biphasic relationship between F-actin polymerization speed and traction force [56], and showed force fluctuation in focal adhesions (FAs) [13]. 

Lately, super-resolved TFM microscopes have also been developed by increasing the accuracy in localizing fluorescent markers embedded in the substrate [15]. An improved fluctuation-based super-resolution (FBSR) traction force microscopy was reported by further increasing the bead density and only embedding fluorescent beads on the topmost layer of the gel [16]. Moreover, the FBSR algorithm and two-step deconvolution led to a better localization of the fluorescent beads. These methods further improved the accuracy in producing the strain map that is important for the reconstruction of the cell adhesive force map with higher resolution. However, of note, the accuracy of bead tracking is different from the resolution of cell adhesive force imaging. Although these methods gained higher accuracy in tracking the beads, the cell adhesive force usually cannot be reconstructed at a resolution comparable to the accuracy level. The computation of high-resolution traction force also demands considerably higher computation power and longer calculation time.

### 2.2. Fluorescent Tension Sensors Bring the Resolution of Force Imaging to Diffraction Limit

In the recent decade, FTSs have been developed to convert cell adhesive force to fluorescence onsite. Immobilized on surfaces, these sensors are equipped with ligands that bind to target force-transmitting receptors (mainly integrins) on the cell membrane. Cells are plated and cultured on sensor-grafted surfaces. The integrins bind and transmit force to the sensors, which respond with a fluorescent signal. While a variety of biomaterial molecules have been adopted to construct these sensors, as detailed in another section, these sensors share a similar force-sensing principle. The main constructs of the sensors are deformable molecules that are responsive to molecular tensions. The constructs are labeled with a dye, a dye–quencher pair or a dye–dye pair. When the construct configuration is altered by force, the fluorescent activity of the sensor is consequently changed. Thus, cell adhesive force can be reported by surface fluorescence loss, fluorescence gain or Förster resonance energy transfer (FRET) [62] efficiency change. Note that the responsivity range of the sensor constructs should match the range of the molecular force transmitted by single receptors (such as integrins). Empirically speaking, integrin tension has a broad range varying in 1–100 pN.

With force-to-fluorescence conversion, the spatial resolution of cell adhesive force imaging was immediately brought to the diffraction limit. A series of fluorescent tension sensors such as MTS [43], TGT [21], MTFM sensor [60], TP [24] and ITS [49] have been developed to image cell adhesive forces. These sensors have been routinely applied to visualize cell adhesive forces in fibroblasts, platelets, keratocytes, T cells, etc. (Figure 2D–F). With these sensors, cell adhesive force has become as visible as cell structures, making the force-structure study highly convenient. A detailed discussion about these sensors is included in next section with an emphasis on biomaterials used in the sensor construction.

Tension sensors report force with high resolution, but usually cannot measure the whole cell force. This is because tension sensor activation by force is stochastic at the molecular level, and not all cell adhesive force is transmitted onto sensors. In this regard, the tension sensor cannot replace TFM. However, the perk brought by tension sensors is not only the improved resolution, but also the capability of evaluating the force level of the molecular tension transmitted by single receptors. The molecular construct of the sensor can be tuned to be responsive to a designed force level, enabling the selective visualization of molecular tension in a certain range. Therefore, although these sensors do not evaluate bulk cell adhesive force, they have a unique advantage of assessing the cell adhesive force at the molecular tension level. 

### 2.3. Super-Resolved Cell Adhesive Force Microscopies

With FTSs, it seems naturally feasible to enable force imaging with resolution beyond the diffraction limit by integrating the sensors with super-resolution microscopies. The term “super-resolution” refers to the resolution beyond the diffraction limit of optical microscopy. Among the various techniques enabling super-resolution imaging, stochastic optical reconstruction microscopy (STORM) [63] and photoactivated localization microscopy (PALM) [64] are the representative ones. STORM and PALM share the similar principle of using single molecule imaging and localization to break the diffraction limit. The dyes in an ensemble are sparsely and stochastically turned on and off, to ensure the imaging of these dyes in a molecularly distinguishable manner. The center of the point-spread functions of fluorescence from each dye can be localized with ultra-high accuracy, which reaches the nanometer level. By imaging and localizing these dyes in many consecutive frames, one can assemble the frames and reconstruct the image of the dye-stained sample with a resolution beyond the diffraction limit. Since FTSs are able to convert force to fluorescence, the technical challenge to achieve super-resolution force imaging is about how to report force signals in a sparse and stochastic manner. 

*Computational super-resolution force microscopy.* Computational super-resolution force microscopy was developed with the combination of molecular tension sensors (MTSs) [61] and Bayesian localization microscopy [65]. In this method, cell adhesive force signals are recorded by MTS immobilized on a surface. The fluorescence activated by cell adhesive force is collected in ensemble, not at the molecular tension level. By inducing bleaching and blinking of the dyes in the consecutive frame, one can apply Bayesian analysis to extract spatial information of the sensor activation sites beyond the diffraction limit. It demonstrated the visualization of traction forces within FAs with about 100 nm spatial resolution (Figure 2G). This technique is the first attempt in the field to obtain cell adhesive force in a super-resolution manner. However, Bayesian analysis is computationally demanding, and it is difficult to acquire the cell adhesive force dynamics in real time. The resulting resolution is generally inferior to that provided by super-resolution microscopies based on single molecule localization.

*Super-resolution force microscopy based on single molecule imaging and localization.* By the time of this review, two force microscopies based on molecule localization have emerged: cellular force nanoscopy (CFN) [54] and tPAINT [55]. Both techniques report integrin tensions in a stochastic and sparse manner, so that the fluorescent tension signals are distinguishable in each frame of imaging for the purpose of molecular localization. Single molecule localization is then performed to localize the tension events with ultra-high accuracy. By repeating this process to each frame, a force map with super-resolution is obtained. Although both CFN and tPAINT use DNA-based tension sensors, there is distinct difference between them in terms of how to achieve sparse and stochastic tension imaging. In CFN, a force-activatable emitter is used as the tension sensor. This emitter is conjugated with a quencher–dye pair, hence being dark until activated by targeted integrin tension. The dye of the emitter is bright and can be easily bleached. As a result, the fluorescence of tension events in each frame is collected and bleached, to ensure the sparsity required by single molecule imaging and localization. Although the emitters are not reusable once activated and photobleached, the high coating density of sensors (~2000/μm^2^) ensures sufficient sensors for cell adhesive force imaging. In tPAINT, the surface-immobilized DNA-hairpin tension sensors are not conjugated with dyes or quenchers. Instead, the sensor has a cryptic site that is openable by force and provides a docking site for complementary ssDNA-dye diffusing in the ambient solution. The sparse and stochastic tension imaging is enabled by the transient binding and dissociation between the docking site and ssDNA-dye. Both CFN and tPAINT have successfully achieved single integrin tension imaging, and demonstrated 50 nm and 25 nm resolutions, respectively, in live cell adhesive force imaging (Figure 2H,I). A potential limit of CFN originates from the irreversible dye bleaching, resulting in a gradual consumption of local sensors by cells. Still, in a practical test, CFN can provide up to 1000 frames of force images before the sensor density has an appreciable decrease. A potential limit of tPAINT could be due to the obstructed or reduced diffusion of ssDNA-dye to sensor location under cell adhesion regions at the cell–substrate interface, therefore affecting the fidelity of reporting local force density. Another technical challenge shared by both CFN and tPAINT is the spontaneous DNA dissociation due to thermal energy which yields non-specific fluorescent signals unrelated to cell adhesive force. In the future, a fluorescent tension sensor with higher thermal stability is desired to reduce or eliminate such background false signals.

## 3. Biomaterial Choices for Constructing Fluorescence Tension Sensors

**Figure 3:**
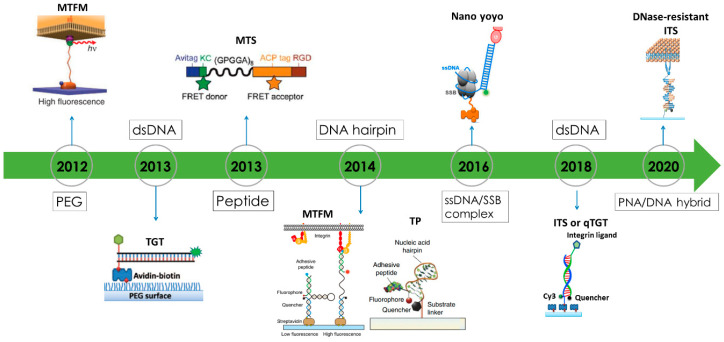
Biomaterials for constructing extracellular fluorescent tension sensors.

As FTSs are in the center of cell adhesive force imaging at high resolution, the critical part of an FTS construct is the force-responsive molecular biomaterial that changes conformation under tension. This section reviews the materials used for tension sensor construction. These materials usually have well characterized mechanical properties and accessible chemistry for molecular engineering. Figure 3 presents these materials adopted in constructing extracellular FTSs in a chronical order. Among these materials, DNA and DNA-like materials are particularly versatile to be used as the constructs of FTSs because of their programmable force sensing thresholds and convenient chemistry for sensor synthesis. 

*Polyethylene glycol (PEG)* as an entropy “spring” was applied in AFM to measure single molecular forces in 1999 [66]. PEG has accessible chemistry for modification, well-characterized mechanical properties and excellent biocompatibility and stability. Wiegand and colleagues reported the use of PEG tension sensors in quantitative analysis of a virus or particles uptake kinetics [39]. Stabley and colleagues constructed a PEG-based tension sensor that is labeled with a dye and immobilized on gold nanoparticle, which functions as a quencher [20]. The sensor successfully reported cell adhesive force by fluorescence turned on by the force. The sensor mapped the mechanical forces during the early stage of regulatory endocytosis of the ligand-activated epidermal growth factor receptor. With the same “spring” molecules conjugated with integrin ligand, Liu and colleagues visualized integrin-transmitted cell adhesive forces at the pN level at the cell–substrate interface. Integrin tension in the range from 1 to 15 pN was reported by quantitative characterization of different PEG molecules [22].

*Peptide* molecules are natural polymers and can function as entropic springs for force sensing. Intracellular tension sensors based on peptide were first developed in 2008 [18]. A peptide in an α-helix conformation coupled with a CFP–GFP pair was developed as a force-sensing cassette and inserted into alpha-actinin, non-erythrocyte spectrin and filamin A. Transfected cells successfully expressed these intracellular tension sensors, which reported cell adhesive force in cells. In 2011, Grashoff and colleagues used repetitive amino-acid motif derived from the spider flagelliform silk protein to replace the α-helix for force sensing [19]. The elastic property of such peptide was characterized by AFM-based force spectroscopy, showing broad force range from several pN to as high as 800–900 pN [67]. The “spring” coupled with two fluorescent proteins as a FRET pair was genetically inserted into a force-bearing protein vinculin to report intracellular force. The vinculin FRET sensor revealed that vinculin was required for stabilizing adhesions under force [19], and reported that tension across vinculin in stable focal adhesions was ~2.5 pN.

Spider silk peptide was also adopted as biomaterial for the construction of an extracellular tension sensor. Morimatsu et al. developed a tension sensor consisting of a (GPGGA)_8_ peptide flanked with two organic dyes as a FRET-pair [23,42,43]. The peptide was decorated with biotin for surface immobilization and RGD peptide as integrin ligand. Integrin tensions were reported by the FRET value of the sensor. The dynamic range of the spider silk peptide-based sensor was 1–5 pN, therefore sensitive to the integrin tension variation at the lower level. In contrast, Galior et al. developed another type of peptide-based tension sensor for reporting integrin tensions at a very high level (>80 pN) [68]. In this design, immunoglobulin 27th (I27) domain of cardiac titin flanked with a fluorophore and gold nanoparticle was used as the main construct of the tension sensor. High-level tension (80−200 pN) [69] is required to expose the cryptic disulfide bonds in this peptide which is cleaved by redox reagent dithiothreitol in the cell culture medium. The disulfide bond cleavage leads to the extension of the peptide and the de-quenching of the fluorophore. As a result, this sensor reports molecular tension higher than 80 pN. It was shown that some integrin tensions are indeed able to activate this sensor, demonstrating the existence of high-level integrin tensions in live cells.

*dsDNA* was initially adopted as a programmable tension sensor to evaluate the strength of molecular bonds such as antigen-antibody bonds [70,71]. Compared to other elastic polymers, DNA enjoys mature chemistry methods for synthesis and modification. Its tension responsivity can be conveniently adjusted by altering the DNA sequence or geometrical configuration (by adjusting the force application sites on the DNA backbones), hence being “programmable”. In 2013, a dsDNA-based tension sensor was first applied to a live cell force study [21]. Tension gauge tether (TGT) with dsDNA as the main construct was developed to quantitatively define the force transmitted by integrin molecules during cell adhesion [21]. TGT can be viewed as a molecular linker with a defined tension tolerance (*T*_tol_). Any integrin tensions higher than the *T*_tol_ value would rupture TGT and the tension becomes abolished, so that molecular tensions are restricted under a designed level. TGT defined the minimum tensions required for integrin and notch activations. Apart from determining the minimum force requirement for a force transmitting receptor activation, a fluorescently labeled TGT was also used to image the distribution of integrin molecular tensions in cells [46,48]. Two distinct levels of integrin tensions were revealed by TGT: ~40 pN integrin tension caused by the cell membrane during initial cell adhesion, and >54 pN integrin tension in FAs due to the actomyosin contraction.

*DNA hairpin* was soon adopted as another DNA construct for tension sensor construction [24,72]. A DNA hairpin is a single-stranded DNA forming intramolecular base pairs. Under a tension, the hairpin can be unzipped but the DNA strand remains intact. When the tension is removed, the hairpin is able to refold back. In contrast to dsDNA, DNA hairpin opening by force is reversible and the hairpin refolding negates the fluorescence signal, hence reporting cell adhesive force in a real-time manner. DNA hairpin-based tension sensors share a similar working mechanism as PEG-based tension sensors. The tension threshold can be more accurately defined by varying the GC content in the DNA sequence [72]. DNA hairpin-based molecular tension sensors were applied to uncover the role of cell adhesive force in cell initial adhesion and T cell activation [72,73]. The force distribution by integrin α_IIb_β_3_ in the process of platelet activation was spatially visualized by DNA hairpin–based tension sensors, revealing that platelets produced a force greater than 19 pN in the central zone but weaker forces (between 4.7 and 13.1 pN) in the cell edge (Figure 2F) [60].

DNA hairpin-based tension sensor was also applied to visualize the adhesive force at the cell-cell interface. Zhao et al. developed a tension sensor with one end labeled by cholesterol tags insertable to cell membrane, and the other end labeled with integrin ligand targeting the integrin, or labeled with cadherin targeting cadherin in neighboring cell surface [44]. This tension sensor successfully reported the force at the cell-cell interface.

*Nano-yoyo* is a tension sensor based on a ssDNA-protein complex that is specialized in reporting low-level molecular tension (~4 pN) [74]. Both dsDNA-based and DNA hairpin-based tension sensors cannot report tensions lower than the DNA unzipping force which is at ~10 pN. In contrast, it was shown that the unbinding force between ssDNA and the SSB protein (ssDNA binding protein) is as low as ~4 pN. This ssDNA-SSB complex has a configuration similar to a yoyo, as the ssDNA wraps on the SSB with several turns, hence the name. The unique property of nano-yoyo is its good thermal stability. This is because of the long contact length between ssDNA and the SSB. In one complex, there are 65 nucleotides of DNA wrapping around the SSB tetramer, producing good thermal stability despite the low unbinding force, as the dissociation energy is determined by the product of force and length. A nano-yoyo-based tension sensor was applied to fathom the force required for Notch activation with higher sensitivity. It showed that the force requirement for Notch activation is as low as 4 pN, updating the previously reported value of <12 pN [21].

*dsDNA labeled with a dye-quencher pair.* Inspired by TGT, dsDNA labeled by a fluorophore-quencher pair, termed as integrative tension sensor (ITS) [49] or quenched TGT (qTGT) [47], was developed to image cell adhesive force with a positive fluorescence signal. The quenched dye on the ITS becomes fluorescent after the upper DNA strand with a quencher is removed by integrin tension, hence reporting cell adhesive force onsite (Figure 1D). The mechanical dissociation of dsDNA on the surface is irreversible, and therefore an ITS remains fluorescent after the force is released, leading to signal integration over time. The accumulated force signal offers higher sensitivity in cell adhesive force imaging, with the trade-off of non-real-time force imaging. However, real-time force mapping can still be achieved by recording the force map in a time-series manner and obtaining the newly produced force signal with the frame subtraction method. Figure 2E demonstrates the real-time force in a migrating keratocyte [50]. ITSs have been applied to study cell adhesive force in keratocytes and platelets. It was shown that high-level integrin tension (>54 pN) was generated at the cell rear margin to detach integrins off from the substrate, therefore facilitating cell rear retraction during fast cell migration [50]. ITSs also revealed the polarized force distribution in adherent platelets [49]. The spatial resolution of cell adhesive force imaging by ITS was calibrated to be 0.3–0.4 μm, approaching the diffraction limit of fluorescence microcopy. 

*PNA/DNA hybrid* was adopted to construct a tension sensor resisting the degradation from DNase on the cell membrane [75]. Many cancer cells such as mouse thyroid carcinoma cells and MDA-MB-231 breast cancer cells were reported to express substantial membrane-bound DNase [76]. We also observed significant DNase activity on the cell membrane of macrophages and neutrophils (unpublished data). Such membrane-bound DNase easily destroys DNA-based tension sensors immobilized at the cell–substrate interface and separating the fluorophore-quencher pair without force involved. Thus, DNA-based tension sensors are potentially problematic in investigating adhesive forces of cancer cells and immune cells. In order to report cell adhesive force without disruption from DNase, Zhao et al. constructed tension sensors with PNA (peptide nucleic acid)/DNA hybrid duplexes [75]. The PNA/DNA hybrid structure shows strong resistance to DNase and retains the ability of reporting cell adhesive force. Moreover, PNA also enhances the fluorescence intensity of cyan dye by two-fold due to the PIFE effect (protein induced fluorescence enhancement), therefore increasing the signal-to-noise ratio in cell adhesive force imaging. PNA/DNA hybrid was used instead of PNA/PNA duplex (which likely has equal resistance to DNase if not better) because the DNA strand is more cost-effective and convenient for dye, quencher or integrin ligand conjugation. The PNA/DNA-based tension sensor broadens the application of FTSs in the study of cell adhesive force in the DNase-present environment. 

## 4. Outlook

Structural and biochemical studies in cells have historically preceded the force study because approaches are more available for visualizing and quantifying structural and biochemical signals. However, just like investigating how a car runs, one not only needs to know the parts constituting the car, but also needs to know how these parts synergistically produce and transmit the force driving the car. Similarly, force visualization and quantification are essential for the study of cell mechanobiology. Techniques were in demand to reveal when and where cell adhesive force is produced at cell–cell and cell–matrix interfaces.

This review summarizes the recent development of techniques for cell adhesive force imaging, with a focus on the advances of spatial resolution and sensor constructs. TFM has pioneered the cell adhesive force visualization and measurement. Even now, it is advantageous in measuring the bulk cell forces, obtaining the local force direction and being applicable in 3-dimensional (3D) force mapping. In comparison, an FTS converts cell adhesive force to fluorescence onsite, greatly improving resolution and sensitivity in force imaging. FTS-based CAFM is also more convenient to implement in terms of assay preparation and data processing. Moreover, an FTS can selectively report receptor-transmitted molecular tensions at different force levels, as the tension sensor has pre-calibrated responsivity to molecular tensions. Overall, TFM and FTSs should be viewed as complementary techniques in cell adhesive force studies. However, in terms of spatial resolution, FTSs with onsite force-to-fluorescence conversion have apparent advantages in achieving high resolution and high sensitivity in cell adhesive force imaging. With FTSs, at present, cell adhesive force has become as visible as cell structures, and the force can even be imaged at the single molecular tension level. Currently, although no research has demonstrated simultaneous monitoring of both adhesive force and structural/biochemical signal at the molecular level in real time, there is no technical barrier in achieving such a feat. The comparable sensitivities and resolutions in cell adhesive force imaging and structure imaging provide great opportunities for the study of force–structure interplay in cells. We speculate that such force–structure imaging at the single molecule level would yield unprecedented insights into the mechanism of mechanotransduction in cells.

The next challenge in cell adhesive force imaging is to generalize FTSs to the 3-dimensional (3D) context. To our knowledge, so far all FTSs were only used on 2D surfaces for imaging cell–matrix forces. There is great difficulty in applying current FTSs directly to 3D cell adhesive force imaging. This is because FTSs generally have non-zero fluorescence background, as the sensors are labeled with dyes (albeit in the proximity of quenchers). The fluorescence background is tolerable if FTSs are coated on 2D surfaces as a molecular monolayer. If grafted in the 3D matrix gel for 3D force imaging, the FTSs in the bulk gel would likely produce a bright background that submerges the cell force signal. We speculate that quenchers with extremely high quenching efficiency would be required for the application of FTSs in the 3D context. Another challenge in the application of FTS sensors comes from their spontaneous activation due to thermal energy. The molecular sensors can stochastically overcome the energy barrier, therefore changing the sensor conformation and non-specifically activating the sensors, transiently or permanently. Such spontaneous activation causes false force signals which is especially problematic for the detection of single molecular tension events at a low rate of occurrence at the cell–matrix interface, such as the tethering force transmitted by selectin during leukocyte rolling, or the force transmitted by the von Willebrand factor receptor during platelet activation, etc. Therefore, lowering the fluorescence background and reducing the non-specific activation rate of FTSs would further improve the performance of FTSs in cell adhesive force imaging, especially at the molecular tension level.

Until now, CAFM was mainly applied to image integrin-transmitted cellular force. Certainly, the central reason is that integrins are the major proteins mediating cell-matrix adhesion and playing vital roles in many cellular functions. In addition, integrins are clustered and form patterns in various cell adhesive units such as focal complexes, focal adhesions, filopodia, podosomes and invadosomes, making force imaging necessary to reveal the spatial dynamics of the integrin-transmitted force. Another reason is perhaps that integrins have a short peptide ligand (peptide RGD consisting of arginine, glycine, and aspartate) which is chemically stable and can tolerate the ligand-tension sensor conjugation process which could be chemically harsh to protein ligands. The small size of the integrin peptide ligand also facilitates FTS immobilization at a high density on a surface. Collectively, these factors make it attractive and feasible to image integrin-transmitted force in cells by FTSs. Still, as the force sensing module of FTSs is seeing steady advancement, the ligand part can use a better variety so that FTSs can be applied to the study of many other mechano-sensitive receptors on the cell membrane.

## Figures and Tables

**Table 1:** Force detection ranges and spatial resolutions of cell adhesive force microscopies.

		Detection Range	Spatial Resolution	References
TFM		~1 nN	1–5 μm	[7,34,35,36,37,38]
Super-resolution TFM		<1 nN	40–80 nm *	[15,16]
FTS-based cell adhesive force microcopy	PEG	0–20 pN	<1 μm	[22,23,39,40,41]
	peptide	1–100 pN	<1 μm	[42,43]
	DNA hairpin	7–14 pN	<1 μm	[24,40,44,45]
	dsDNA	12–54 pN	<1 μm	[46,47,48,49,50,51,52,53]
CFN		12–54 pN	50 nm	[54]
tPAINT		7–14 pN	25 nm	[55]

* Not force resolution, but accuracy of bead tracking.

## Abbreviations

CAFM	Cell adhesive force microscopy
CFN	Cellular force nanoscopy
FTS	Fluorescent tension sensor
ITS	Integrative tension sensor
MTFM	Molecular tension fluorescence microscopy
MTS	Molecular tension sensor
TFM	Traction force microscopy
TGT	Tension gauge tether
TP	Tension probe
t-PAINT	tension-point accumulation in nanoscale topology
